# Attitudes toward withholding antibiotics from people with dementia lacking decisional capacity: findings from a survey of Canadian stakeholders

**DOI:** 10.1186/s12910-021-00689-1

**Published:** 2021-09-06

**Authors:** Gina Bravo, Lieve Van den Block, Jocelyn Downie, Marcel Arcand, Lise Trottier

**Affiliations:** 1 Department of Community Health Sciences, Faculty of Medicine and Health Sciences, Université de Sherbrooke, Sherbrooke, Canada; 2 Research Centre On Aging, CIUSSS de l’Estrie – CHUS, 1036 South Belvedere Street, Sherbrooke, J1H 4C4 Canada; 3 VUB-UGhent End-of-Life Care Research Group, Vrije Universiteit Brussel (VUB), Brussels, Belgium; 4 Schulich School of Law and Faculty of Medicine, Dalhousie University, Halifax, Canada; 5 Department of Family Medicine, Faculty of Medicine and Health Sciences, Université de Sherbrooke, Sherbrooke, Canada

**Keywords:** Dementia, Decisional incapacity, Advance directive, Infection, Antibiotics, Continuous deep sedation, Euthanasia, Medical assistance in dying (MAID), Survey, Canada

## Abstract

**Background:**

Healthcare professionals and surrogate decision-makers often face the difficult decision of whether to initiate or withhold antibiotics from people with dementia who have developed a life-threatening infection after losing decisional capacity.

**Methods:**

We conducted a vignette-based survey among 1050 Quebec stakeholders (senior citizens, family caregivers, nurses and physicians; response rate 49.4%) to (1) assess their attitudes toward withholding antibiotics from people with dementia lacking decisional capacity; (2) compare attitudes between dementia stages and stakeholder groups; and (3) investigate other correlates of attitudes, including support for continuous deep sedation (CDS) and medical assistance in dying (MAID). The vignettes feature a woman moving along the dementia trajectory, who has refused in writing all life-prolonging interventions and explicitly requested that a doctor end her life when she no longer recognizes her loved ones. Two stages were considered after she had lost capacity: the advanced stage, where she likely has several more years to live, and the terminal stage, where she is close to death.

**Results:**

Support for withholding antibiotics ranged from 75% among seniors and caregivers at the advanced stage, to 98% among physicians at the terminal stage. Using the generalized estimating equation approach, we found stakeholder group, religiosity, and support for CDS and MAID, to be associated with attitudes toward antibiotics.

**Conclusions:**

Findings underscore the importance for healthcare professionals of discussing underlying values and treatment goals with people at an early stage of dementia and their relatives, to help them anticipate future care decisions and better prepare surrogates for their role. Findings also have implications for the scope of MAID laws, in particular in Canada where the extension of MAID to persons lacking decisional capacity is currently being considered.

**Supplementary Information:**

The online version contains supplementary material available at 10.1186/s12910-021-00689-1.

## Background

Lower respiratory tract infections, particularly pneumonia, are common in nursing home residents at later stages of dementia, increasing the risk of discomfort and death in this already frail population1[1–4]. When a person who lacks decisional capacity contracts a life-threatening infection, the decision to initiate or withhold antibiotic therapy most often falls to surrogate decision-makers, typically close family members assisted by healthcare professionals [5, 6]. Such decisions can be difficult to make, for several reasons.

First, some patients will not have made their wishes for health care known in advance of loss of capacity [7–10]. Others may have discussed their wishes with their loved ones, and some may have put them in writing, but without being specific about how they want to be treated if they were to develop a life-threatening infection [10]. Second, the extent to which antibiotic therapy significantly improves comfort or prolongs life in these patients is still being debated in the scientific literature [11–13]. Third, antibiotic therapy may be burdensome and cause suffering in itself, often requiring hospitalisation, parenteral administration, adequate hydration for greater efficacy, and restraints to prevent removal of intravenous lines [11, 14, 15]. Concerns about antimicrobial resistance complicate things further [13, 16–18], especially in long-term care settings where most people at later stages of dementia are cared for and will ultimately die [19, 20]. Unless a clear and well-informed advance directive^1^ is available, choosing the best approach to life-threatening infections can thus be challenging, requiring surrogate decision-makers and healthcare professionals to carefully balance complex medical and ethical considerations (e.g., whether antibiotics are medically indicated or futile in the situation; cost-utility issues; whether to accept potential burden caused by treatment; weighing the patient’s best interests against patient and family preferences; balancing benefit to the present patient given antibiotics against harm to future patients due to antimicrobial resistance) [16, 21–23].

The widely-endorsed principle of respect for patient autonomy would require decision-makers to follow the patient’s goals of care, when these are known and apply to the clinical situation. However, a multitude of factors besides previously expressed wishes have been shown to influence decisions regarding antibiotic therapy, both in practice and when studied through hypothetical scenarios [23–29]. Potentially influential factors include clinical features of the infectious episode; patients’ current condition (e.g., illness severity, perceived quality of life, prognosis); families’ wishes and beliefs; expected treatment effectiveness and impact on patients’ quality of life; ethical considerations as those listed above; and characteristics of care providers (e.g., age, gender, religiosity, training in end-of-life care). Antibiotic use has also been shown to vary across countries, due to differences in legislation, care organization, culture, and staff competences in palliative care [23, 30–33].

Past research has thus uncovered determinants of people’s attitudes toward starting or withholding antibiotic therapy in the presence of dementia. However, as far as we know, no study has directly compared attitudes across dementia stages and stakeholder groups. Moreover, attitudes toward other options for relieving suffering at the end of life, specifically continuous deep sedation (CDS) and medical assistance in dying (MAID), have rarely been investigated for their associations with attitudes toward the use of antibiotics in the context of dementia.

CDS relieves suffering from refractory symptoms by rendering the patient unconscious until death, whereas MAID involves ending the patient’s life through a lethal injection [34]. CDS is considered an ethically acceptable means to relieve suffering when death is imminent, although acceptability is reduced by the possible life-shortening effects of this medical procedure in situations where artificial nutrition and hydration is forgone [35, 36]. In patients with dementia and pneumonia, CDS may enhance comfort in the days before death [2, 34, 37]. Still, there is no consensus about the appropriateness of using CDS in advanced dementia patients, whose inability to participate in decision-making makes assessing the intensity of their suffering a challenging task for physicians [38]. MAID is an even more controversial practice, especially in later stages of dementia [39]. At the time the present study was conducted, only three countries (the Netherlands, Belgium and Luxembourg) allowed formerly capable patients to access MAID through a prior written request, provided that they meet the legal requirements.

In Canada, at the time of our study, MAID was legal for those who met the eligibility criteria and procedural safeguards set out in the Criminal Code [40]. Some Canadians with dementia (even as their sole underlying medical condition) could qualify for MAID and some received MAID [41–44]. To be eligible they had to have decision-making capacity at the time of provision of MAID and have met the eligibility criteria.^2^,^3^ However, many Canadians (including healthcare professionals) were unaware of this and believed that MAID was entirely inaccessible to people with dementia – wrongly believing that someone with dementia could not both have decision-making capacity and meet the eligibility criteria for MAID.

In the Canadian province of Quebec where our study was conducted, except in emergency situations, if the patient is incapable of giving or refusing consent to care and has not drawn up advance medical directives, a person authorized by law or by a protection mandate^4^ may do so in the patient’s place. In the absence of advance medical directives,^5^ consent is given by his or her court-appointed representative (a mandatory, tutor or curator). If the patient is not so represented, consent is given by someone close to the patient (i.e., his or her spouse or partner, an involved relative or friend). The person consenting on behalf of the patient (herein called the surrogate decision-maker) must act in the sole interest of that patient, complying, as far as possible, with any wishes the latter may have expressed [49]. If the incapacitated patient has issued advance medical directives, the healthcare provider is not required to obtain authorization from the patient’s surrogate decision-maker. Advance medical directives are binding, which means that healthcare providers who are aware of their existence must comply with them (provided the care is medically indicated in the clinical situation).

Given rapidly changing practices in end-of-life care, and gaps in current knowledge on attitudes toward antibiotics for people with dementia lacking decisional capacity, this study seeks to answer the following three questions: (1) To what extent do different stakeholders support withholding antibiotics from people at later stages of dementia?; (2) Does support vary between dementia stages and stakeholder groups?; and (3) Are stakeholders’ sociodemographic characteristics and support for CDS and MAID associated with their attitudes toward antibiotics? Based on past findings [16, 23, 25, 50], we expected lay respondents to be less in favour of withholding antibiotics than healthcare professionals. Additionally, although the effect of dementia stage has not been studied explicitly [23], we expected support for withholding antibiotics to be higher for people closer to death. Conflicting results regarding the influence of respondent sociodemographic characteristics on end-of-life decision-making, and the paucity of studies that have investigated support for CDS and MAID as potential correlates, precluded formulating a priori hypotheses regarding the associations of these factors with attitudes toward antibiotics in the presence of dementia [24, 27, 51–53].

## Methods

### Study design, target populations, and sampling

The data originate from an anonymous vignette-based survey designed to elicit the views of four groups of Quebec stakeholders (senior citizens, family caregivers, nurses and physicians) on end-of-life care options in the presence of dementia. Details of the survey have been published elsewhere [54]. Briefly, the four population-specific surveys were conducted consecutively from September 2016 to December 2017. Survey packages were mailed to 621 community-dwelling adults aged 65 and over, randomly sampled from the provincial health insurance database. Family caregivers (*n* = 471) were reached through regional Alzheimer Societies, whereas random lists of nurses (*n* = 514) and physicians (*n* = 653) likely to be involved in dementia care were provided by their professional associations. Sample sizes were established a priori, based on expected participation rates [54]. Respondents could provide their answers on the paper version of the questionnaire or access it on online using a single-use, personal access code. Following reminders sent two and nine weeks after the first mailing, 1184 of the 2259 sampled individuals returned the survey questionnaire. Excluding 134 persons who self-identified as non-eligible, the overall response rate was 49.4% (seniors 54%, caregivers 69%, nurses 59%, physicians 25%).

### Survey questionnaire

The questionnaire began with the list of the eligibility criteria for MAID, and ended with questions aimed at describing survey participants. In between was a series of vignettes designed to elicit respondents’ attitudes toward end-of-life care options (see Additional file 1: Survey items used in this paper). The vignettes feature a 75-year-old hypothetical woman, Mrs. Jackson, who has recently been diagnosed with Alzheimer disease. Following discussions with her treating physician and close relatives, Mrs. Jackson has documented her wishes for when she is no longer able to make healthcare decisions for herself. In writing, she has refused all life-prolonging interventions and explicitly requested that a doctor end her life when she no longer recognizes her loved ones. In the next vignettes, Mrs. Jackson, now lacking decisional capacity, has evolved to the advanced and then to the terminal stage of her illness. At the advanced stage, she is described as likely having several more years to live, while at the terminal stage, she is believed to be at the end of her life. At both stages, respondents were asked to rate the extent to which they found it acceptable (1) that the current legislation be changed to allow a doctor to grant Mrs. Jackson’s request for MAID, and (2) that antibiotics be withheld from Mrs. Jackson should she develop a life-threatening infection. At the terminal stage only, respondents were further asked their attitudes toward deeply sedating the patient continuously until death to relieve her persistent distress. Responses were provided on a 5-point scale, ranging from *totally unacceptable* to *totally acceptable*.

### Statistical analysis

Respondent characteristics and attitudes are summarized using means, standard deviations or percentages, depending on the measurement scale. To ensure adequate cell sizes for comparative analyses, answers to attitudinal items were dichotomized, comparing respondents who considered a practice to be acceptable in a given scenario (*somewhat* or *totally*) with those who did not. We used the generalized estimating equation (GEE) approach to investigate whether dementia stages, stakeholder groups, sociodemographic characteristics, and support for CDS and MAID, were related to attitudes toward antibiotics [55]. Modelling began by testing the statistical significance of the *group-by-stage* interaction to determine whether subsequent analyses had to be conducted separately for the advanced and terminal stages of dementia. Reported *p*-values are two-sided. GEE results are described using odds ratios (ORs) and 95% confidence intervals (CIs). Data analysis was carried out using IBM SPSS for Windows, version 25.

## Results

### Respondent characteristics and support for MAID and CDS

Respondent characteristics measured in all four stakeholder groups, and support for MAID and CDS in the context of dementia, are shown in Table 1. Average age ranges from 49 years among physicians to 73 among seniors. Women outnumber men in all four groups, with percentages ranging from 53% among seniors to 82% among nurses. Reflecting the gradual secularization of the province of Quebec since the 1960s, average scores on the index of religiosity were relatively low [56], especially among physicians. Between 60 and 70% of respondents had cared for a dying relative or friend, and less than half had recorded their own healthcare wishes in the event of incapacity. As previously reported [57], at the terminal stage, support for extending MAID to the patient was relatively high in all four groups, from 71% among physicians to 91% among family caregivers, and always higher than at the advanced stage. At the terminal stage, support for MAID was higher than for CDS among seniors, caregivers and nurses, whereas no difference was found among physicians. In other words, Canadian stakeholders reported being less comfortable with a currently lawful end-of-life practice (CDS) than with one that is not (MAID in the context of decisional incapacity). Although not the topic of this paper, this finding has implications for MAID laws which, ultimately, should reflect societal values [29].

**Table 1:** Respondent characteristics and attitudes toward continuous deep sedation and medical assistance in dying for a person with dementia who lacks decisional capacity^a^

Characteristics and attitudes	Seniors *n* = 317	Caregivers *n* = 306	Nurses *n* = 291	Physicians *n* = 136
Age (in years)	72.7 ± 5.9	65.9 ± 11.4	51.7 ± 9.1	49.2 ± 12.5
Gender (female)	53%	72%	82%	60%
Religiosity^b^	5.9 ± 4.2	5.9 ± 4.0	5.3 ± 3.6	3.5 ± 3.5
Have accompanied a dying relative or friend through the dying process	70%	67%	69%	60%
Have themselves completed an advance directive	44%	43%	34%	49%
Find it acceptable (*somewhat* or *totally*) to extend medical assistance in dying to the person with dementia				
At the advanced stage	76%	68%	53%	45%
At the terminal stage	90%	91%	83%	71%
Find it acceptable (*somewhat* or *totally*) to deeply sedate the person with dementia until death, at the terminal stage	79%	69%	70%	68%

^a^Data shown are means ± standard deviations or percentages derived from valid cases. Few data were missing: from 1 for gender to 23 for one or more of the four items involved in deriving the religiosity index

^b^The religiosity index is derived by combining answers to four questions developed by Statistics Canada for the General Social Survey [56]. Total scores range from 0 to 13 and are interpreted in three broad categories: low (0 – 5), moderate (6 – 10), and high (11 – 13)

### Attitudes toward withholding antibiotics, and correlates

**Fig. 1:**
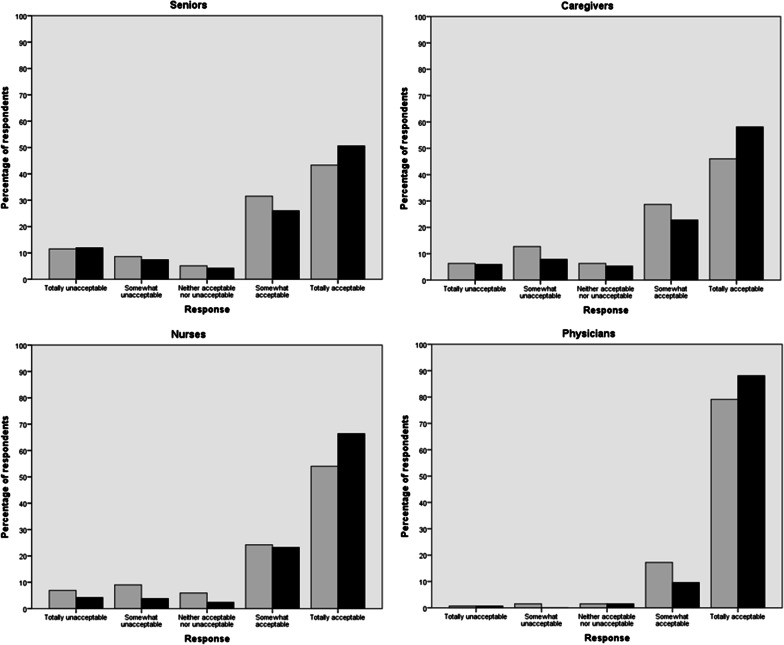
Acceptability of withholding antibiotics from a person with dementia who lacks decisional capacity, at the advanced (in grey) and terminal (in black) stages, by stakeholder group

Figure 1 shows the extent to which respondents found it acceptable to withhold antibiotic therapy from the depicted patient, stratified by stakeholder group and dementia stage. In the context where the patient has refused in writing all medical interventions that could prolong her life after she is no longer able to make health-related decisions (see Additional file 1: Survey items used in this paper), the acceptability of withholding antibiotics was relatively high in all four stakeholder groups. Combining *somewhat acceptable* and *totally acceptable* responses, support ranged from 75% among seniors and caregivers at the advanced stage, to 98% among physicians at the terminal stage. Visual inspection of Fig. 1 suggests some effect of the dementia stage, which was confirmed by a statistically significant *group-by-stage* interaction (*p* = 0.003). Accordingly, subsequent analyses were carried out separately for the advanced and terminal stages.

Table 2 shows the results of investigating the associations between various factors and respondents’ attitudes toward withholding antibiotics from the depicted patient, at the advanced stage of dementia. No differences in support for withholding antibiotics were detected between caregivers and seniors, nor between nurses and seniors, while the odds of physicians’ supporting this practice were 15 times higher than among seniors (*p* < 0.001). Gender and level of religiosity were both found to be associated with attitudes toward withholding antibiotics, but not age, having accompanied a dying loved one, or having an advance directive. Compared to men, women had 1.5 higher odds of supporting this practice, whereas a 5-point decrease in the index of religiosity (pointing to less religious respondents) increases by 27% the odds of finding it acceptable to withhold antibiotics from the patient (OR = 1.27). Attitudes toward allowing MAID at the advanced stage of dementia were also associated with the outcome. Proponents had 3.5 higher odds of finding it acceptable that antibiotics be withheld, compared to opponents.

**Table 2:** Multivariable GEE results from investigating factors related to finding it acceptable (*somewhat* or *totally*) to withhold antibiotics from a person with dementia who lacks decisional capacity, at the advanced stage

	**OR**	***p*-value**	**95% CI**
*Group* effect			
Senior citizens (reference)	1		
Family caregivers	0.98	0.914	0.6 – 1.5
Nurses	1.6	0.115	0.9 – 2.8
Physicians	15.1	** < 0.001**	4.9 – 46.4
Age	1.0	0.858	0.98 – 1.02
Gender			
Male (reference)	1		
Female	1.5	**0.017**	1.1 – 2.2
Religiosity	0.95	**0.032**	0.9 – 1.0
Have accompanied a dying relative or friend through the dying process	1.1	0.515	0.8 – 1.6
Have themselves completed an advance directive	1.3	0.153	0.9 – 1.8
Find it acceptable (*somewhat* or *totally*) to extend medical assistance in dying to the person with dementia, at the advanced stage			
No (reference)	1		
Yes	3.5	** < 0.001**	2.5 – 5.0

Bold is used to highlight p-values that are smaller than 0.05

At the terminal stage (cf. Table 3), nurses and physicians were more likely than seniors to support the withholding of antibiotics (OR = 3.0, *p* = 0.003, and OR = 28.2, *p* < 0.001, respectively), with no difference found between caregivers and seniors. In the context of terminal dementia, religiosity is the only sociodemographic characteristic found to be associated with attitudes toward the use of antibiotics. Support for MAID and support for CDS are also associated with the outcome, with positive attitudes toward these practices increasing the odds of being in favour of withholding antibiotics (OR = 3.2 and 5.2, respectively, *p* < 0.001). Accounting for other influential factors, the odds of supporting the withholding of antibiotics from a person with dementia who lacks decisional capacity is thus higher among stakeholders who also support extending MAID to this population (Tables 2 and 3) or relying on CDS to relieve the person’s suffering when death is imminent (Table 3).

**Table 3:** Multivariable GEE results from investigating factors related to finding it acceptable (*somewhat* or *totally*) to withhold antibiotics from a person with dementia who lacks decisional capacity, at the terminal stage

	**OR**	*p*-value	95% CI
*Group* effect			
Senior citizens (reference)	1		
Family caregivers	1.4	0.157	0.9 – 2.3
Nurses	3.0	**0.003**	1.5 – 6.0
Physicians	28.2	** < 0.001**	6.0 – 132.5
Age	1.0	0.260	0.97 – 1.01
Gender			
Male (reference)	1		
Female	1.2	0.513	0.8 – 1.8
Religiosity	0.93	**0.004**	0.9 – 1.0
Have accompanied a dying relative or friend through the dying process	0.86	0.475	0.6 – 1.3
Have themselves completed an advance directive	1.4	0.124	0.9 – 2.1
Find it acceptable (*somewhat* or *totally*) to extend medical assistance in dying to the person with dementia, at the terminal stage			
No (reference)	1		
Yes	3.2	** < 0.001**	1.9 – 5.4
Find it acceptable (*somewhat* or *totally*) to deeply sedate the person with dementia, at the terminal stage			
No (reference)	1		
Yes	5.2	** < 0.001**	3.4 – 7.9

Bold is used to highlight p-values that are smaller than 0.05

## Discussion

To the best of our knowledge, this study is the first to investigate variability across dementia stages and stakeholder groups in the acceptability of withholding antibiotics from a person with dementia who lacks decisional capacity, in the context where she has refused all life-prolonging interventions and explicitly requested that a doctor end her life when she no longer recognizes her loved ones. Faced with this decision, the vast majority of our respondents would likely decide against initiating antibiotics (cf. Figure 1). Still, substantial minorities, mainly among senior citizens, family caregivers and nurses, found withholding antibiotics unacceptable, even at the terminal stage and with some knowledge of the patient’s wishes. Like others [51, 53], we found religiosity to exert some influence on attitudes toward end-of-life care, with the least religious of our respondents being more likely to support withholding antibiotics from the patient, at both stages of dementia. We also found positive attitudes toward MAID and CDS to increase the likelihood of finding it acceptable to withhold antibiotics. Regarding MAID, our finding concurs with those of Hinkka et al*.* [51], who found, among Finnish physicians, support for “active euthanasia” to be associated with a preference to withhold antibiotics from a terminally ill cancer patient.

The person featured in our vignettes wanted to avoid prolonged dementia, either by the withholding of a potentially life-prolonging intervention or by MAID (should it be legalized). According to our findings, which revealed substantial variability in the acceptability of withholding antibiotics from the now-incapacitated person, whether her wish is honored could depend on who has decision-making authority for or influence over medical decisions. This variability can be explained by several factors.

First, although not unusual, Mrs. Jackson’s instructions for future care are not explicit about antibiotic therapy for pneumonia, requiring some interpretation. Most physicians and a significant number of nurses, perhaps more knowledgeable about antibiotics, may have interpreted the person’s refusal of all life-prolonging interventions to include antibiotic therapy, while many senior citizens and family caregivers may not have seen antibiotics as life-prolonging. Lay respondents’ answers to the vignettes may have differed had Mrs. Jackson been more specific. Forms for accepting or refusing medical interventions for times of incapacity, such as advance medical directives in Quebec, would benefit from expressly addressing antibiotic therapy, given the high prevalence of infections in later stages of dementia. Having observed that support for end-of-life practices varies between dementia stages, such forms could also be more dementia-specific. This could be achieved by describing the main dementia stages (e.g., mild, moderate, severe) and allowing the person to choose different options of care for each of these stages [58, 59]. Better still, because documentation of wishes for end-of-life care without communication may not be effective, wishes should be discussed within advance care planning (ACP)^6^ conversations between the person with dementia, their relatives and healthcare professionals [61, 62].

Second, respondents’ answers may have been influenced by a projection bias, which leads people to choose care options that reflect what they would want for themselves rather than preferences previously expressed by the person who now lacks decisional capacity [63, 64].

Third, at the advanced stage, where the patient is depicted as likely having several more years to live and not being in distress, some respondents may have felt that her life was still worth living, prioritizing what they believed was in her best interests at the time rather than her previously expressed wishes. At the advanced stage, the percentages of senior citizens and family caregivers against withholding antibiotics are similar to those against extending MAID to the person with dementia (cf. Table 1). This reinforces the impression that some respondents were not comfortable with a decision that would hasten death at this stage of the dementia trajectory and in the absence of distress.

Such considerations are less likely to explain lay respondents’ reluctance to withhold antibiotics at the terminal stage, where the person was depicted as being at the end of her life and in distress. Here, not recognizing dementia as a terminal condition, or not viewing pneumonia as part of a “natural death” for someone with severe dementia, are plausible explanations. In focus groups with U.S. family members of nursing home residents with moderate to very severe dementia, Forbes et al. [50] found that in the absence of a clear understanding of the dying trajectory, family members felt compelled to treat “treatable conditions” such as pneumonia, even in late-stage dementia. Fear of abandoning the patient and of making the wrong decision have also been cited by families as reasons for providing treatment that professionals may consider futile [25, 52, 65].

### Implications for practice

The explanations proposed above underscore the importance of explaining the risks, burdens and benefits of care options to patients and their family members, and exploring patients’ underlying values and preferences for comfort or life-prolonging care [66]. Initiated in the earlier stages of dementia, these ACP conversations would aim at helping the patient make fully-informed decisions regarding future care, preparing surrogate decision-makers for their role (which could include deciding whether antibiotics should be initiated or withheld) and increasing the likelihood that the patient’s wishes will later be honored [61, 62]. A booklet developed in Canada for informing families on the course of dementia, possible complications, therapeutic options and decision-making (e.g., regarding antibiotics) could prove useful in achieving these objectives [67]. Available in several languages, the booklet could also be used as a complementary educational tool for healthcare professionals [68, 69]. As this booklet targets family members and not persons with dementia, it does not contain a form to document wishes for times of incapacity. However, ACP booklets with sample plans are available in several countries and languages [e.g., 70, 71].

### Limitations and strengths

Our study has limitations that warrant comment. First, response rates and sample sizes are relatively large, except for physicians. As a result, ORs are less precise for this stakeholder group. Moreover, cell size considerations led us to collapse response options for analysis, thereby losing some information. Larger samples would allow more refined analyses. Second, family caregivers of persons with dementia who are not members of Alzheimer Societies could not be reached through our recruitment strategy. Moreover, as for any survey, individuals who elected to complete the questionnaire may differ from non-respondents. In particular, our samples may have included people with a keen interest in MAID, whether in favour or opposed. MAID was a hot topic in Canada at the time of the survey, and still is. Third, findings are based on samples from Quebec, potentially affecting generalizability, especially to jurisdictions with different legislation governing end-of-life care for incapacitated patients. Fourth, vignettes are valuable for comparing opinions on controversial topics, but they are limited in the amount of information they can convey. In real-life situations, decision-makers would likely have more knowledge of the patient than that provided in a fictitious case description. Despite being defined in the survey questionnaire, the terms *advanced* and *terminal* that we used to describe dementia stages may have been interpreted differently by some respondents. Fifth, respondents were asked to rate the acceptability of different care options, and not what decision they would have made in the depicted situations. The vignettes featured a third party, and not a loved one or themselves. We do not know whether framing the questions differently would have changed our findings. Also, the decision to provide or withhold antibiotics for a life-threatening pneumonia as inferred from respondents’ acceptability levels could differ from the decision that would actually be made in practice, as the latter would likely benefit from healthcare professionals’ and family members’ discussing the benefits and burdens of the various care options [72]. Lastly, the survey was conducted 4 years ago, and end-of-life care practices evolve rapidly. A follow-up survey would be worth doing, as well as focus groups to deepen our understanding of stakeholders’ preferences for one practice over others.

Our study also has strengths. These include the random selection of potential respondents belonging to relevant stakeholder groups; the use of the same set of vignettes across groups, enabling direct comparisons of their views; attitudes explored at two distinct stages along the dementia trajectory; and analyses that included respondents’ attitudes toward other end-of-life medical interventions, in addition to their sociodemographic characteristics.

## Conclusions

Using a fictitious person with dementia who had refused in writing all life-prolonging interventions and requested MAID, we found relatively high support for withholding antibiotics in the event of a life-threatening infection, but also variability across stakeholder groups. Physicians consider withholding antibiotics more acceptable than other stakeholders. Higher support for this practice was also found among less religious respondents and those supporting MAID and CDS in the context of dementia. Professionals caring for patients with dementia should acknowledge differences in attitudes toward end-of-life care options, which may complicate decision-making at the end of life. Discussions about end-of-life care goals and options, including the use of possibly life-prolonging treatments such as antibiotics, with patients and their loved ones early in the dementia trajectory could help people with dementia make informed choices about future care and clearly communicate their wishes about such care, in addition to helping surrogate decision-makers to honour those wishes.

## Supplementary Information

**Additional file 1.** Survey items used in this paper.

## Data Availability

The dataset used for the current paper is available from the corresponding author on reasonable request.

## Abbreviations

CDS: Continuous deep sedation
MAID: Medical assistance in dying
GEE: Generalized estimating equation
OR: Odds ratio
CI: Confidence interval
ACP: Advance care planning

## Footnotes

1 In this paper, an advance directive (whether legally binding or not) is any document (e.g., a living will) in which a person expresses his or her values, preferences, or goals of care in the event that he or she should become decisionally incapacitated. Such document aims at helping those who could be called upon, in the future, to make health-related decisions on the person’s behalf.

2 The eligibility criteria were:

- they have a serious and incurable illness, disease, or disability;
- they are in an advanced state of irreversible decline in capability;
- that illness, disease, or disability or that state of decline causes them enduring physical or psychological suffering that is intolerable to them and that cannot be relieved under conditions that they consider acceptable; and
- their natural death has become reasonably foreseeable, taking into account all of their medical circumstances, without a prognosis necessarily having been made as to the specific length of time that they have remaining.

3 After the study was completed, the Canadian law was amended, removing the requirement that the person’s natural death is reasonably foreseeable and allowing a form of advance request for MAID [45]. If a person met the first three eligibility criteria set out above and made a written agreement for the provision of MAID on a specific date in the future, they could receive MAID after losing decision-making capacity.

4 A protection mandate is a document in which a person can choose one person or several people (called a mandatory or mandatories) to make decisions on his or her behalf in the event of incapacity and give instructions about health care that he or she wants or does not want [46].

5 Advance medical directives are another way of expressing one’s wishes regarding medical care in the event of incapacity [47]. Created under the Act Respecting End-of-Life Care that legalized MAID in certain circumstances [48], advance medical directives have priority over a protection mandate if both exist and the incapacitated patient is in one of the clinical situations in which they apply: i.e., the patient (1) suffers from a serious and incurable medical condition, and is at the end of life; (2) is in a coma deemed irreversible or in a permanent vegetative state; or (3) has severe dementia with no possibility of improvement. For each situation, the person can accept or refuse five interventions: cardiopulmonary resuscitation, ventilator-assisted breathing, dialysis, and artificial feeding and hydration. For greater access, advance medical directives are filed in a registry held by the Quebec health insurance board (Régie de l’Assurance maladie du Québec). Medical assistance in dying cannot be asked for in advance medical directives.

6 ACP is “a process that supports adults at any age or stage of health in understanding and sharing their personal values, life goals and preferences regarding future medical care” [60]. ACP can include conversations with people likely to be involved in their future care (e.g., treating physician, close relatives) and documentation of values and preferences for care.
