# Supplementary material for: Attitudes toward withholding antibiotics from people with dementia lacking decisional capacity: findings from a survey of Canadian stakeholders
Source: BMC Med Ethics. 2021 Sep 6;22:119. doi: 10.1186/s12910-021-00689-1 (PMC8420012; doi:10.1186/s12910-021-00689-1)
Supplement: Supplementary file 1 — Additional file 1. Survey items used in this paper. [file 12910_2021_689_MOESM1_ESM.docx]

**Extracts from the questionnaire assessing attitudes toward care options for a person with dementia who lacks decisional capacity, at the advanced and terminal stages**

Mrs. Jackson is a 75-year-old retired teacher who was diagnosed with dementia three months ago. A few years earlier, Mrs. Jackson’s mother had died with dementia. Although Mrs. Jackson knows that her quality of life could be good in the early stages, she fears losing her capacity to care for herself. She knows that there is currently no cure for her disease.

Together with her loved ones and doctor, Mrs. Jackson discusses the health care that she would or would not want to receive in the future. Next, she records her wishes in a document. In this document, she refuses all medical interventions that could prolong her life after she is no longer able to make health-related decisions. She also explicitly requests that a doctor end her life when she no longer recognizes her loved ones.

She gives copies of her document to her loved ones. In the following years, she often reminds them and her doctor of the wishes she expressed in the document. Her loved ones agree to speak up to ensure her wishes are respected.

**Advanced stage**

Six years later, Mrs. Jackson, now 81, cannot take care of herself anymore. She now lives in a long-term care home and is no longer able to make health-related decisions. However, she does not seem uncomfortable and likely has many more years to live. She interacts well with other residents and her relatives when they visit but does not seem to know who they are anymore.

As instructed by Mrs. Jackson, her loved ones show her doctor the document in which she asked that a doctor end her life when she could no longer recognize her loved ones.

**1.** *To what extent would you find it acceptable for the Government of Quebec to change the current legislation to allow a doctor to administer to Mrs. Jackson, at the advanced stage of her disease, a substance that would cause her death in a matter of minutes?*

| Totally unacceptable | Somewhat unacceptable | Neither acceptable nor unacceptable | Somewhat acceptable | Totally acceptable |
| --- | --- | --- | --- | --- |
|  |  |  |  |  |

**2.** Suppose that, at the advanced stage of her disease, Mrs. Jackson contracts an infection that could be fatal. Antibiotic treatment for the infection could prolong Mrs. Jackson’s life by several months or even a few years.

*Knowing that Mrs. Jackson refused all life-prolonging interventions in her advance request, to what extent would you find it acceptable for the physician to refrain from administering antibiotics to treat her infection, while optimizing her comfort and relieving her pain?*

| Totally unacceptable | Somewhat unacceptable | Neither acceptable nor unacceptable | Somewhat acceptable | Totally acceptable |
| --- | --- | --- | --- | --- |
|  |  |  |  |  |

**Terminal stage**

Mrs. Jackson is still unable to make health-related decisions and her physical health has seriously deteriorated. According to her doctor, she has reached the terminal stage and only has a few weeks left to live. She is no longer able to feed herself and must now be spoon-fed. For some time now, she has been showing signs of distress. She cries a lot daily, even when surrounded by her loved ones. All efforts to alleviate her pain and anxiety have failed.

Mrs. Jackson’s loved ones remind her doctor of the document in which she asked that a doctor end her life when she could no longer recognize her loved ones.

**3.** *To what extent would you find it acceptable for the Government of Quebec to change the current legislation to allow a doctor to administer to Mrs. Jackson, at the terminal stage of her disease, a substance that would cause her death in a matter of minutes?*

| Totally unacceptable | Somewhat unacceptable | Neither acceptable nor unacceptable | Somewhat acceptable | Totally acceptable |
| --- | --- | --- | --- | --- |
|  |  |  |  |  |

**4.** Suppose that, at the terminal stage of her disease, Mrs. Jackson contracts an infection that could be fatal. Antibiotic treatment for the infection could prolong Mrs. Jackson’s life by a few weeks.

*Knowing that Mrs. Jackson refused all life-prolonging interventions in her advance request, to what extent would you find it acceptable for the physician to refrain from administering antibiotics to treat her infection, while optimizing her comfort and relieving her pain?*

| Totally unacceptable | Somewhat unacceptable | Neither acceptable nor unacceptable | Somewhat acceptable | Totally acceptable |
| --- | --- | --- | --- | --- |
|  |  |  |  |  |

**5.** Suppose Mrs. Jackson had not asked for a physician to end her life when she became totally incapable of recognizing her loved ones. Faced with Mrs. Jackson’s persistent distress, her loved ones ask the physician to put her into a deep, uninterrupted sleep. Given that this would not be accompanied by artificial feeding or hydration, Mrs. Jackson would likely die within a few days.

*To what extent would you find it acceptable, at the terminal stage of Mrs. Jackson’s disease, for a physician to put her gradually into a deep, uninterrupted sleep, knowing that Mrs. Jackson would likely die within a few days?*

| Totally unacceptable | Somewhat unacceptable | Neither acceptable nor unacceptable | Somewhat acceptable | Totally acceptable |
| --- | --- | --- | --- | --- |
|  |  |  |  |  |
